# Sex and age correlations of reported and estimated physical fitness in adolescents

**DOI:** 10.1371/journal.pone.0219217

**Published:** 2019-07-03

**Authors:** Lovro Štefan, Petar Paradžik, Goran Sporiš

**Affiliations:** 1 Department of General and Applied Kinesiology, Faculty of Kinesiology, University of Zagreb, Zagreb, Croatia; 2 ‘VII’ High-school, Zagreb, Croatia; Teesside University/Qatar Metabolic Institute, UNITED KINGDOM

## Abstract

The main purpose of the study was to explore sex and age specific correlations between self-reported and estimated physical fitness. In this cross-sectional study, we recruited 1 036 secondary-school students (55.3% girls). Self-reported physical fitness was assessed on a 10-point scale, with a higher score indicating better physical fitness. We measured waist circumference, sit-ups in 1 minute, standing long jump and sit-and-reach test and calculated *z*-scores to obtain objective physical fitness index. Boys performed better in sit-ups in 1 minute and standing long jump tests and had higher waist circumference values. Girls performed better in sit-and-reach test. Overall, boys had higher physical fitness *z*-score values, compared to girls. Younger boys had better physical fitness perception (*r*_1st grade_ = 0.61, *p*<0.001), compared to older ones (*r* = 0.40–0.50, *p*<0.001). In girls, correlation coefficient was the highest in the 3^rd^ grade (*r* = 0.46, *p*<0.001), followed by the 2^nd^grade (*r* = 0.43, *p*<0.001), the 1^st^grade (*r* = 0.41, *p*<0.001) and the 4^th^ grade (*r* = 0.37, *p*<0.001). Our study shows moderate sex and age specific correlation between self-reported and estimated physical fitness in a large sample of adolescents.

## Introduction

Physical fitness is considered one of the most important health markers [1] and a significant predictor of all-cause mortality [2]. It is defined as ‘the capacity to perform physical activity to a full range of physiological and psychological qualities’ [1]. The level of physical fitness in children and adolescents is associated with health in adulthood [3], pointing out that timely screening of and specific interventions to promote physical fitness may support disease prevention.

Components of physical fitness include: (1) cardiorespiratory, (2) musculoskeletal, (3) motor and (4) body composition fitness [3]. All components can be objectively assessed, often through laboratory or field-based testing [4]. Disadvantages of objective estimates of physical fitness, including cost and time of implementation, can limit their practical application in population based studies.

Another way to estimate physical fitness is through self-reported perception. It is a cost-and-time effective way to collect the data. Previous studies have tried to examine the correlation and agreement between self-reported and objectively estimated physical fitness in youth [4,5] and the general population [6–9]. Findings of those studies have been inconsistent, showing both negative [5], and positive [4,6–10] associations between the two variables. The inconsistency comes from using different components of objectively estimated physical fitness. Also, self-reported questions reflecting the level of physical fitness were not the same in aforementioned studies.

Although adolescents represent a population of apparently healthy individuals, the level of physical fitness established in adolescence often persists later in life [3], showing the importance of this parameter on overall health. Moreover, field-based physical fitness tests have shown reliable and valid properties, yet sometimes it is not possible to conduct a population-based study, due to lack of facilities, shortage of researchers and equipment. Therefore, self-reported measures often become more practical and easy to use. Therefore, the main purpose of the study was to explore sex and age specific correlations between self-reported and estimated physical fitness in adolescents.

## Materials and methods

### Study participants

In this cross-sectional study, participants were secondary-school students. At the first stage, we randomly selected 11 (8 grammar and 3 vocational) out of 86 secondary-schools currently operating in the city of Zagreb. At the second stage, we randomly selected one class representing each grade within the school (from 1^st^ to 4^th^). Each class had ≈25 students. All students were considered healthy and were not affected by diseases. The selection criteria were: (1) active participation in physical education classes and (2) absence of injuries. According to the Croatian Bureau of Statistics for the year 2017 [11], there were 36 350 secondary-school students in total. Our sample size was estimated to be 1 030 by using 95% confidence level and 3% margin error. At the beginning, we recruited 1 247 participants. Of these, 136 did not provide full data and 75 were absent when the study was conducted. Our final sample was based on 1 036 secondary-school students (m_age_±SD = 16.3±1.1 yrs; m_height_±SD = 1.74±0.1 m; m_weight_±SD = 64.7±12.4 kg; m_body-mass index_±SD = 21.3±3.0 kg/m^2^; 55.3% girls). After the selection of each school and class, we contacted physical education teachers to help us organize the study and obtain the approval of the principal. The measurement protocol for the study lasted from January to March 2019. For ≈25 students, it took us 30 minutes in each physical education class to collect the data. Before the study began, all students had got familiar with aims, hypotheses and benefits for participation in the study. All procedures performed in this study were in accordance with the Declaration of Helsinki and approved by the Institutional Review Board of the leading author. Also, all participants and their parents/guardians provided written informed consent for participation in the study.

### Self-reported physical fitness

Perceived physical fitness was measured with one item: “How would you rate your physical fitness?” ranging from 1 (very poor fitness) to 10 (excellent fitness) [10]. This measure has been correlated with measures of objective physical fitness and perceived well-being [12] and it has been used among young adults in previous studies [13].

### Objectively estimated physical fitness

We used EUROFIT Battery Fitness Test to assess the level of physical fitness in adolescents. These tests are considered reliable and valid instruments to measure the level of physical fitness in children and adolescents [14]. Waist circumference, standing long jump, sit-ups in 1 minute and sit-and-reach test were chosen because of their mutual independence to the other [15]. Data were collected by two trained researches in order to guarantee the standard measurement methodology [15]. A brief explanation of each test is presented below:

Waist circumference: Each participant stood still in a standing position. We used anthropometric tape placed horizontally midway between the lower rib margin and the iliac crest at the end of normal expiration [16].

Standing long jump: Each subject performed distance jumps from a standing start. While performing the jumps, the subjects were asked to bend their knees with their arms in front of them, parallel to the ground, then to swing both arms, push off vigorously and jump forward as far as possible, trying to land with their feet together and stay upright. The best out of two attempts was taken as the final score (expressed in centimetres) [17].

Sit-ups in 1 minute: Trunk strength was assessed as the maximum number of sit-ups achieved in one minute. Children were seated on the floor, backs straight, hands clasped behind their neck, knees bent at 90° with heels and feet flat on the mat. They then lay down on their backs, shoulders touching the mat, and returned to the sitting position with their elbows out in front to touch their knees, keeping the hands clasped behind their neck the whole time. The total amount of correctly performed sit-ups in 60 seconds was the score [17].

Sit-and reach test: Sitting on the floor or a mat, legs straight under the angle of 90º, the person being tested reached forward with the arms (hands overlapping). The distance of reach was measured in centimetres using a measuring non-elastic tape attached on the floor [18].

Height and weight were collected using portable stadiometer (Seca stadiometer and balance beam) with accuracy nearest to 0.5 cm and 0.2 kg in accordance to the International Society for the Advancement of Kinanthropometry [19].

### Data analysis

Data are presented as mean (SD) for normally distributed or median (25–75 interquartile range) for not normally distributed variables. All variables were grouped according to sex (boys vs.girls) and age (1^st^ grade = 15 year old, 2^nd^ grade = 16 year old, 3^rd^ grade = 17 year old and 4^th^ grade = 18 year old). Differences between normally and not normally distributed variables were analyzed using univariate analysis of variance (ANOVA) and Kruskal-Wallis one-way analysis of variance (H-test). Previous studies have shown sex and age differences in perceived and estimated physical fitness [20]. Therefore, sex and age specific correlations between self-reported (independent) and estimated (dependent) physical fitness were calculated using Spearman’s coefficient with 95% confidence interval. First, we calculated correlations between self-reported physical fitness and each physical fitness test. To get overall objectively estimated physical fitness index, we calculated *z*-scores for each physical fitness test. Then, we summed all *z*-score values. Of note, we also checked for multicollinearity between physical fitness tests using the variance inflation factor (VIF). The VIF value was <2 indicating no multicollinearity between physical fitness tests. Significance was set up at *p*≤0.05, and it was two sided (2-sided). All the analysis were performed in Statistical Package for Social Sciences Software, ver. 22 (IBM Corp., Armonk, NY, USA).

## Results

Basic descriptive statistics of the study participants are presented in Table 1. Boys had higher values of waist circumference (p<0.001) and performed better in 1 minute sit-ups test (p<0.001) and standing long jump (p<0.001) compared with girls. However, girls performed better in sit-and-reach test (p<0.001) compared with boys. When *z*-score for each physical fitness test was calculated and summed, boys had significantly higher values of overall physical fitness index (p<0.001). Compared to objectively estimated physical fitness, boys reported higher values of perceived physical fitness (p<0.001) compared with girls. Differences between objectively estimated physical fitness and self-reported physical fitness according to age were not observed (p>0.05).

**Table 1:** Basic descriptive statistics of the study participants, stratified by sex and age (Croatia, 2019).

Study variables	Total sample (N = 1036)	Boys (N = 463)	Girls (N = 573)	p-value
	Mean (SD)	Mean (SD)	Mean (SD)	
**Waist circumference** **(cm)**				
1^st^ grade	72 (8)	75 (8)	70 (8)	
2^nd^ grade	72 (9)	77 (8)	69 (8)	
3^rd^ grade	74 (10)	79 (11)	69 (7)	
4^th^ grade	75 (10)	80 (10)	70 (6)	<0.001
**Sit-ups in 1 minute** **(x)**				
1^st^ grade	49 (12)	55 (14)	46 (10)	
2^nd^ grade	52 (10)	56 (9)	48 (9)	
3^rd^ grade	52 (12)	56 (11)	47 (11)	
4^th^ grade	52 (13)	57 (14)	47 (11)	<0.001
**Standing long jump** **(cm)**				
1^st^ grade	182 (29)	205 (26)	167 (19)	
2^nd^ grade	183 (33)	206 (32)	167 (24)	
3^rd^ grade	193 (35)	215 (29)	167 (21)	
4^th^ grade	188 (33)	211 (28)	166 (21)	<0.001
**Sit-and-reach test** **(cm)**				
1^st^ grade	66 (13)	61 (10)	70 (13)	
2^nd^ grade	70 (14)	64 (13)	73 (13)	
3^rd^ grade	66 (13)	62 (11)	72 (14)	
4^th^ grade	65 (14)	62 (14)	69 (13)	<0.001
Perceived fitness* (1–10 scale)				
1^st^ grade	7 (5–8)	7 (6–9)	6 (4–7)	
2^nd^ grade	7 (5–8)	7 (7–8)	6 (5–8)	
3^rd^ grade	7 (6–8)	7 (6–8)	6 (5–8)	
4^th^ grade	7 (5–7)	7 (6–8)	6 (5–7)	<0.001

*median (25–75 percentile range)

In boys, self-reported physical fitness was not significantly correlated with waist circumference (1^st^ grade r = -0.10, p = 0.300; 2^nd^ grade r = 0.07, p = 0.504; 3^rd^ grade r = -0.11, p = 0.199 and 4^th^ grade r = 0.02, p = 0.875). Other objectively estimated physical fitness tests were significantly correlated with self-reported physical fitness in all grades (sit-ups in 1 minute 0.60, 0.20, 0.40 and 0.51; standing long jump 0.50, 0.25, 0.53 and 0.28; sit-and-reach test 0.37, 0.26, 0.23, 0.36). In girls, self-reported physical fitness was also not significantly correlated with waist circumference (1^st^ grade r = -0.05, p = 0.532; 2^nd^ grade r = -0.09, p = 0.227; 3^rd^ grade r = -0.10, p = 0.292 and 4^th^ grade r = -0.18, p = 0.072). As in boys, other objectively estimated physical fitness tests were significantly correlated with self-reported physical fitness in all grades (sit-ups in 1 minute 0.37, 0.15, 0.40 and 0.34; standing long jump 0.50, 0.51, 0.41 and 0.28; sit-and-reach test 0.15, 0.31, 0.32, 0.35).

**Fig 1:**
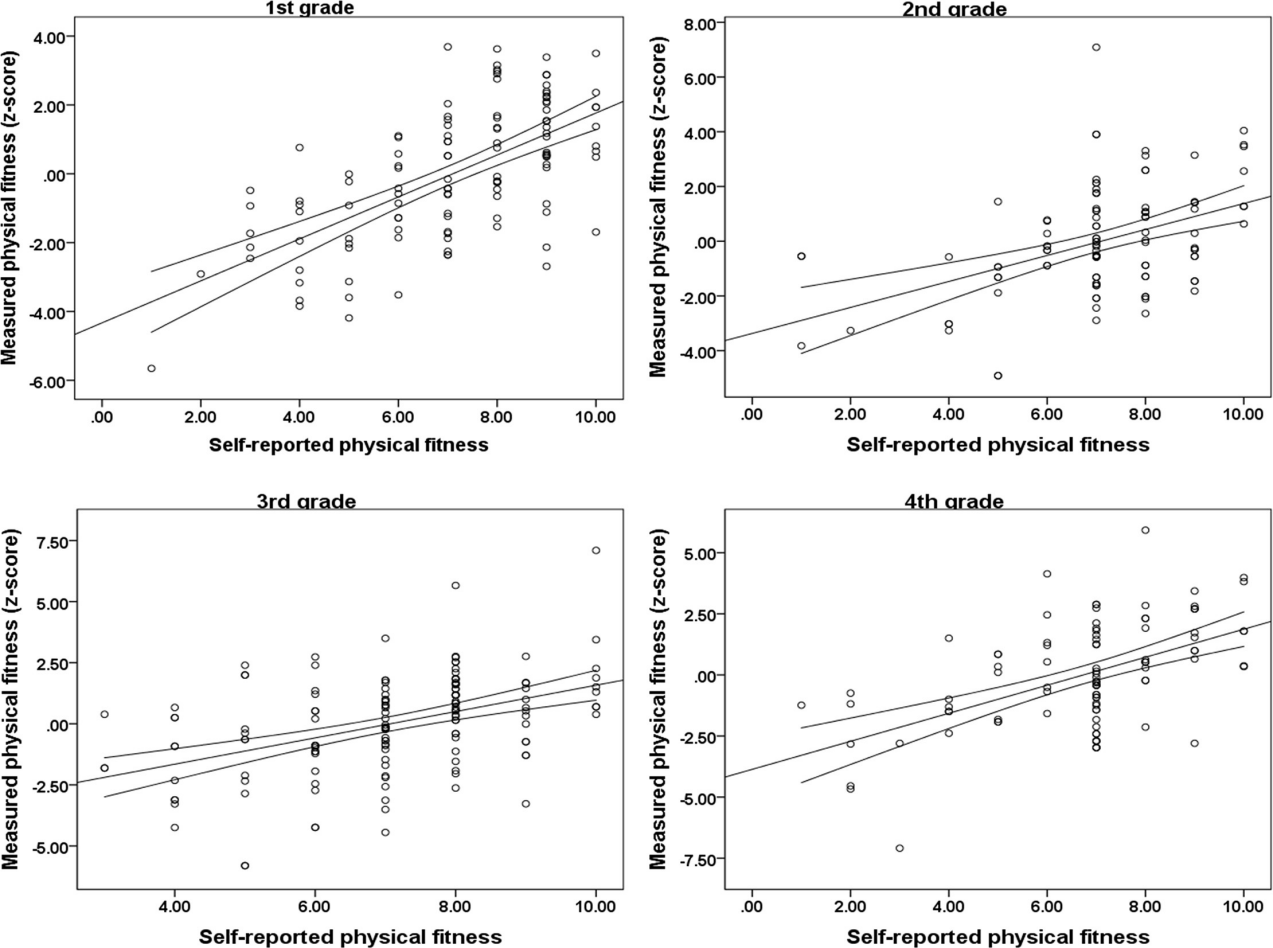
Age specific correlations between self-reported (x-axis) and measured (y-axis) physical fitness in boys (Croatia, 2019).

**Fig 2:**
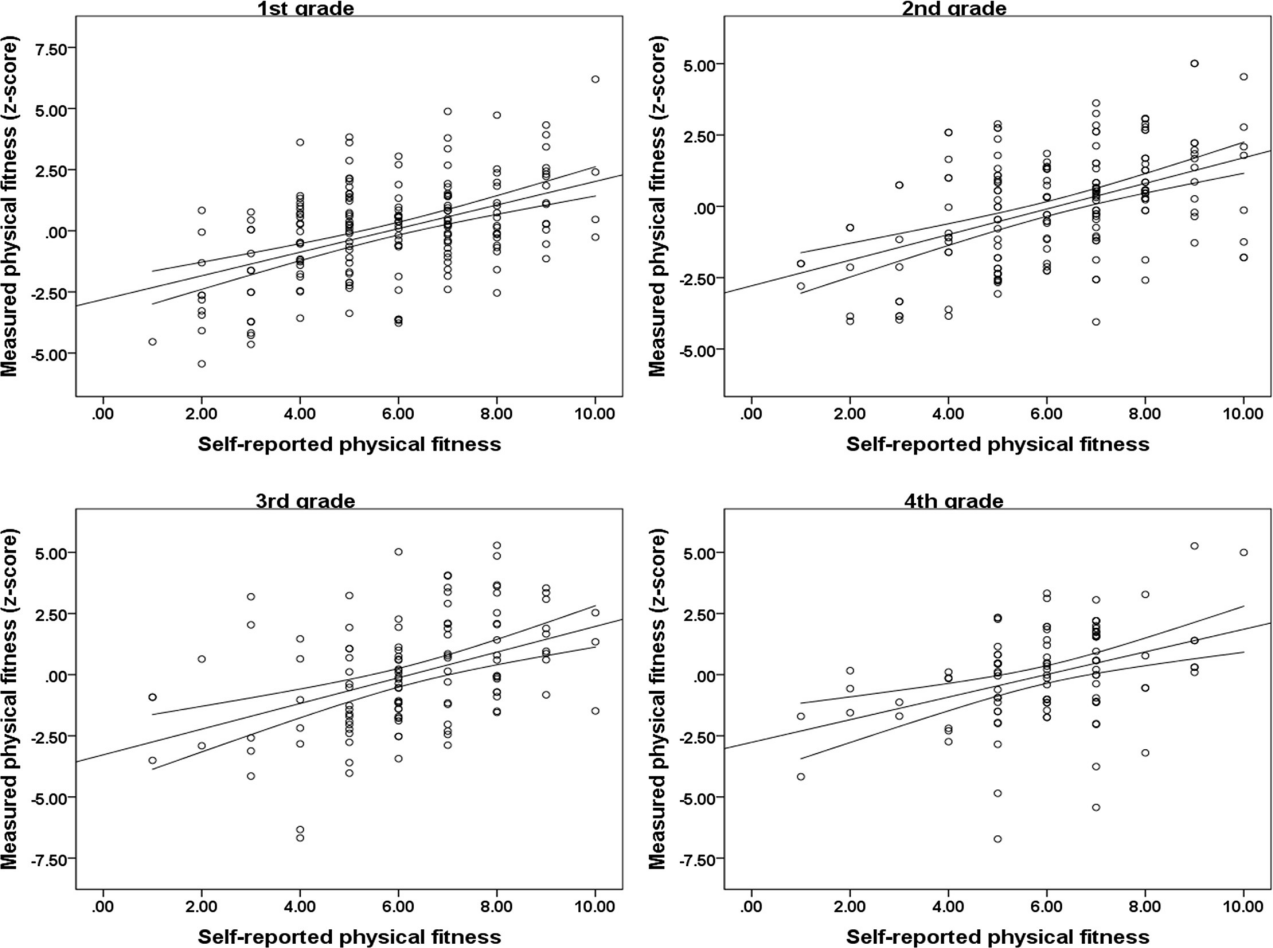
Age specific correlations between self-reported (x-axis) and measured (y-axis) physical fitness in girls (Croatia, 2019).

Sex and age specific correlations of self-reported and estimated overall physical fitness are presented in Fig 1 (boys) and Fig 2 (girls). In boys, the highest correlation between self-reported and objectively estimated physical fitness was observed in the 1^st^ grade (r = 0.61, p<0.001), followed by the 4^th^ grade (r = 0.50, p<0.001), the 3^rd^ grade (r = 0.44, p<0.001) and the 2^nd^ grade (r = 0.40, p<0.001). When boys were observed as a group, the correlation coefficient between self-reported and objectively estimated physical fitness was 0.50 (p<0.001). In girls, the highest correlation between self-reported and objectively estimated physical fitness was observed in the 3^rd^ grade (r = 0.46, p<0.001), followed by the 2^nd^ grade (r = 0.43, p<0.001) and the 1^st^ grade (r = 0.41, p<0.001). The lowest correlation between the aforementioned variables was observed in the 4^th^ grade (r = 0.37, p<0.001). The total correlation coefficient between self-reported and objectively estimated physical fitness in girls was 0.41 (p<0.001)

## Discussion

The main purpose of the study was to explore sex and age specific correlations between self-reported and estimated physical fitness in a large sample of adolescents. Our study shows moderate correlation between the two variables in both boys and girls and according to age.

Our results are similar to one previous study exploring the same correlations in youth [4]. The significant and moderate correlation coefficient in boys (r = 0.50) and girls (r = 0.41) from our study was comparable to previous research including Ortega et al. (r = 0.54 to 0.65) [4], but contrasted with one study focusing on adolescent girls (Young, r = -0.52 to -0.56) [5]. This difference may be due to the fact that we included a larger sample of participants (N = 1 036 vs. N = 193) and used different physical fitness components (musculoskeletal and body composition fitness compared to cardiorespiratory fitness). Furthermore, Ortega *et al*. [21] found a moderate agreement between self-reported (the ‘International Fitness Scale’) and measured (20-m shuttle run test, hand-grip test, the standing long jump test and the sit and reach test) physical fitness in 10 to 13 year old Spanish children ranging from 0.54–0.65. Studies conducted among a general population presented findings similar to our results [6–9]. All of them reported moderate significant correlation between self-reported and estimated physical fitness. Of note, most of the studies used only cardiorespiratory fitness [6,7,9] as a proxy of overall fitness, pointing out that by including other components of physical fitness (musculoskeletal, motor and body composition), correlations might have been different. On the other hand, Mikkelsson *et al*. [22] used more complete tests for overall physical fitness; that is the index of estimated physical fitness was calculated by summing up *z*-scores of a submaximal bicycle ergometer test, ergojump tests, a 30-s sit-up test, hand-grip test and a sit-and-reach test. The same study showed a significant correlation between self-reported and objectively estimated physical fitness (*r*_both sexes_ = 0.54), concluding that at group level their study participants estimated the level of physical fitness moderately well [22]. Strøjer et al. [23]on the other hand used visual analogue scales and illustrations with three motor (muscle strength, flexibility and balance) and one functional (endurance) fitness tests to explore the correlation with objectively estimated physical fitness (assessed with Aastrand test, maximal isometric voluntary contraction of the back extension and flexion muscles, the finger to floor method, isometric back extension and sitting on a wobble board). They found that self-reported physical fitness correlated moderately with aerobic fitness (*r* = 0.36–0.64), muscle strength (*r* = 0.30–0.51) and flexibility (*r* = 0.31–0.36) [23].

Physical fitness is a multidimensional construct with several components taken into account [4]. Although previous studies have shown moderate correlation between self-reported and objectively estimated physical fitness in youth [4,5,21] and general population[6–9,22,23], comparisons and conclusion drawn from the findings should be made with caution, due to a great heterogeneity in terms of sample size, chronological age, questions used to assess self-reported physical fitness and tests measured to assess objective physical fitness. Therefore, a single-item question to assess the level of self-reported physical fitness is not perhaps the best method to measure overall physical fitness in clinical settings [9]. However, in population-based and health-related studies among adolescents, self-reported measure may serve as an easy tool for potential screening and detecting individuals who are at extreme risk of low physical fitness level. Future studies should use standardized methodology (equal single/multi-item questions and physical fitness tests) in order to generate comparable data between different countries and populations.

Our study has several limitations. First, by using a cross-sectional design, we cannot conclude the causality of the correlation. Second, to assess objectively estimated physical fitness, we only used musculoskeletal and body composition components, taking cardiorespiratory fitness out of the equation. As mentioned above, most of the previous studies have used cardiorespiratory fitness as a proxy of overall physical fitness and shown moderate correlation with self-reported measure [6,7,9,21].By using cardiorespiratory fitness in our study, it is possible that we would have obtained somewhat higher correlation coefficients. However, a most recent meta-analysis of longitudinal studies has revealed moderate-large negative association between muscular fitness in childhood/adolescence and adiposity and cardiometabolic parameters in adulthood, pointing out that muscle-strengthening activities have beneficial effects on health during lifespan [24]. Third, we only used a one-item question ranging from 1 to 10 to assess the level of physical fitness. Although, it has been used in previous studies [10,12,13], it does not capture separate components of physical fitness in adolescents, like the ‘International Fitness Scale’ [4].Finally, we did not collect some other potential factors that previous studies have shown to might influence the aforementioned correlations, such as residential area, parenting styles, parents’ activity levels and children’s self-esteem [20].

## Conclusions

Our study shows moderate correlation between self-reported and estimated physical fitness in a large sample of Croatian adolescents. If such measure is used in clinical settings or population-based studies conducted among adolescents, objectively estimated fitness accounts for 25% and 17% of the variance in boys and girls self reported fitness respectively.

## Supporting information

S1 Raw Data (XLSX)
